# The relationship between shyness and depression: the multiple mediating roles of sense of security and adaptability

**DOI:** 10.3389/fpsyg.2026.1690111

**Published:** 2026-01-28

**Authors:** Yuxia Wang, Jiayan Zhang, Yixiu Cong

**Affiliations:** 1 School of Marxism Studies, Shandong Women’s University, Jinan, Shandong, China; 2 Shunhua School, Jinan, Shandong, China; 3 Faculty of Psychology, Shandong Normal University, Jinan, Shandong, China

**Keywords:** adaptability, college students, depression, sense of security, shyness

## Abstract

The overall prevalence of depression among Chinese college students has currently reached 28.4% and continues to rise annually. However, most current research focuses on adolescents, with less attention given to depression among college students. According to the diathesis-stress model, depression results from both individual vulnerability factors and stressful events. Therefore, this study aims to investigate college students (*M*_*age*_ = 19.82, SD = 0.98; Female: 76.8%) through a questionnaire method and analyze the potential mechanisms linking shyness, sense of security, adaptability, and depression using a multiple mediation model. The study found that: (1) shyness significantly and positively predicted depression; (2) sense of security mediated the relationship between shyness and depression; (3) adaptability mediated the relationship between shyness and depression; (4) sense of security and adaptability jointly mediated the relationship between shyness and depression. This study reveals the mechanisms linking shyness to depression in college students, specifically the mediating roles of sense of security and adaptability. This not only enriches the diathesis-stress model but also offers new insights into reducing depression among college students.

## 1 Introduction

In recent years, the number of students diagnosed with mental illness in China has increased, with a younger age of onset (Fu et al., 2023). A meta-analysis revealed that the overall prevalence of depression among Chinese college students has reached 28.4% and continues to rise annually (Gao L. et al., 2020; Wang et al., 2020). Depression negatively impacts individuals’ lives (Formica et al., 2023), academic performance (Deng et al., 2022), and can even lead to self-harm and suicidal behaviors (Zhao et al., 2021). College students are in the transition from late adolescence to early adulthood, a period marked by numerous challenges and an increased risk of setbacks. This makes them a high-risk group for depression (Wang et al., 2024). However, current research on depression primarily focuses on adolescents, often overlooking the prevalence among college students. Therefore, exploring the mechanisms of depression among college students and developing strategies to prevent and reduce its occurrence is urgent.

### 1.1 Shyness and depression

According to the diathesis-stress model, depression results from a combination of individual vulnerability factors and stressful events. Diathesis includes personality, cognitive, and physiological factors, while stressful events include chronic stressors, internal stressors, and major life events (Ma et al., 2018; Mineka et al., 2020). Consequently, individuals predisposed to vulnerability are more likely to develop it when exposed to stressful stimuli. A substantial body of research has supported the universality and reliability of this theory, including gene-environment studies, large cohort studies, and experimental research (Colodro-Conde et al., 2018; Su et al., 2022; Farre et al., 2024). Shyness, a key personality trait, refers to the tendency to feel embarrassed, nervous, or anxious in social situations, often due to a perception of being judged by others (Henderson and Zimbardo, 2001). Previous studies have shown that shyness significantly predicts depression in college students, with individuals exhibiting higher levels of shyness being more prone to depression (Gao F. Q. et al., 2020; Geng et al., 2021). According to the diathesis-stress model, individuals with shyness are at greater risk of depression when exposed to stressful stimuli. This may be due to shy individuals having lower self-esteem, which can lead to maladaptive responses to stress (Liu et al., 2025). For example, shy college students may view themselves as inadequate when confronted with academic opportunities, which may lead them to give up. As a result, they are more likely to experience negative emotions and develop depression (Xu et al., 2024). Based on this, we propose Hypothesis 1: Shyness positively predicts depression, such that higher levels of shyness are associated with greater depression.

### 1.2 Shyness and depression: mediating roles of sense of security and adaptability

A sense of security refers to an individual’s anticipation of psychological or physical danger and their perceived ability or inability to cope with it (Cong and An, 2004). Research shows that a sense of security is a protective factor and negatively predicts depression (Fan et al., 2021). Individuals with a high sense of security are better equipped to cope with stressful events, reducing their negative impact (Jiang and Li, 2020; Li et al., 2022). However, shy individuals often have a low sense of security (Li et al., 2022). Compared to non-shy individuals, shy individuals tend to have lower self-evaluations and are more likely to anticipate poor performance in upcoming situations. This fear-driven expectation reduces their sense of security (Gao et al., 2016). Furthermore, a chronic sense of insecurity can lead to negative emotions, making sense of insecurity a persistent source of stress (Obrenovic et al., 2021; Jandaghian-Bidgoli et al., 2024). Specifically, shy college students often fear poor performance in activities such as class participation or extracurricular engagements. Moreover, this constant anticipation amplifies their fear, keeping them in a prolonged state of insecurity. According to the diathesis-stress model, when individuals face stressful stimuli, their vulnerability (shyness) and stress (sense of insecurity) interact, increasing their risk of depression. Thus, we propose Hypothesis 2: Sense of security mediates the relationship between shyness and depression. Specifically, higher shyness levels are associated with lower sense of security and increased depression.

Adaptability refers to an individual’s ability to adjust themselves or their environment during socialization, thereby improving their interaction with the environment (Zhang and Zhang, 2007). It primarily includes aspects such as learning, interpersonal relationships, daily life, environment, occupation, and physical and mental well-being (Lu, 2003). Research shows that shy individuals are more prone to maladaptation (Yang et al., 2015). Chronic maladaptation, a persistent stressor, not only affects individuals’ interpersonal and academic outcomes (Huang et al., 2022), but is also strongly associated with depression (Horgan et al., 2016; Ota et al., 2020). Specifically, the university years represent a transitional phase into society, during which individuals must adapt to their environment, enhance their skills, and cultivate the ability to maintain interpersonal relationships. However, shy individuals often struggle to achieve these goals, which makes them more prone to maladaptation. Based on the diathesis-stress model, we propose Hypothesis 3: Adaptability mediates the relationship between shyness and depression. Specifically, individuals with higher shyness levels tend to have lower adaptability and higher depression levels.

Additionally, research shows a significant positive correlation between a sense of security and adaptability (Wu et al., 2024). Shy individuals often feel overwhelmed and uncomfortable in unfamiliar or unpleasant situations (Royal et al., 2018), leading to feelings of insecurity and difficulty adapting to their environment (Liu et al., 2019; Geng et al., 2021). Specifically, shy college students often have fear-driven expectations about their performance in unfamiliar situations, which typically results in a lower sense of security and further impedes their ability to adapt to the current environment. According to the diathesis-stress model, the interaction between vulnerability factors and stressors increases individuals’ susceptibility to depression. Over time, this accumulation can lead to depression (Colodro-Conde et al., 2018). Thus, we propose Hypothesis 4: Sense of security and adaptability play multiple mediating roles in the relationship between shyness and depression. Specifically, higher shyness levels are associated with lower sense of security, lower adaptability, and increased depression.

### 1.3 The present study

**FIGURE 1:**
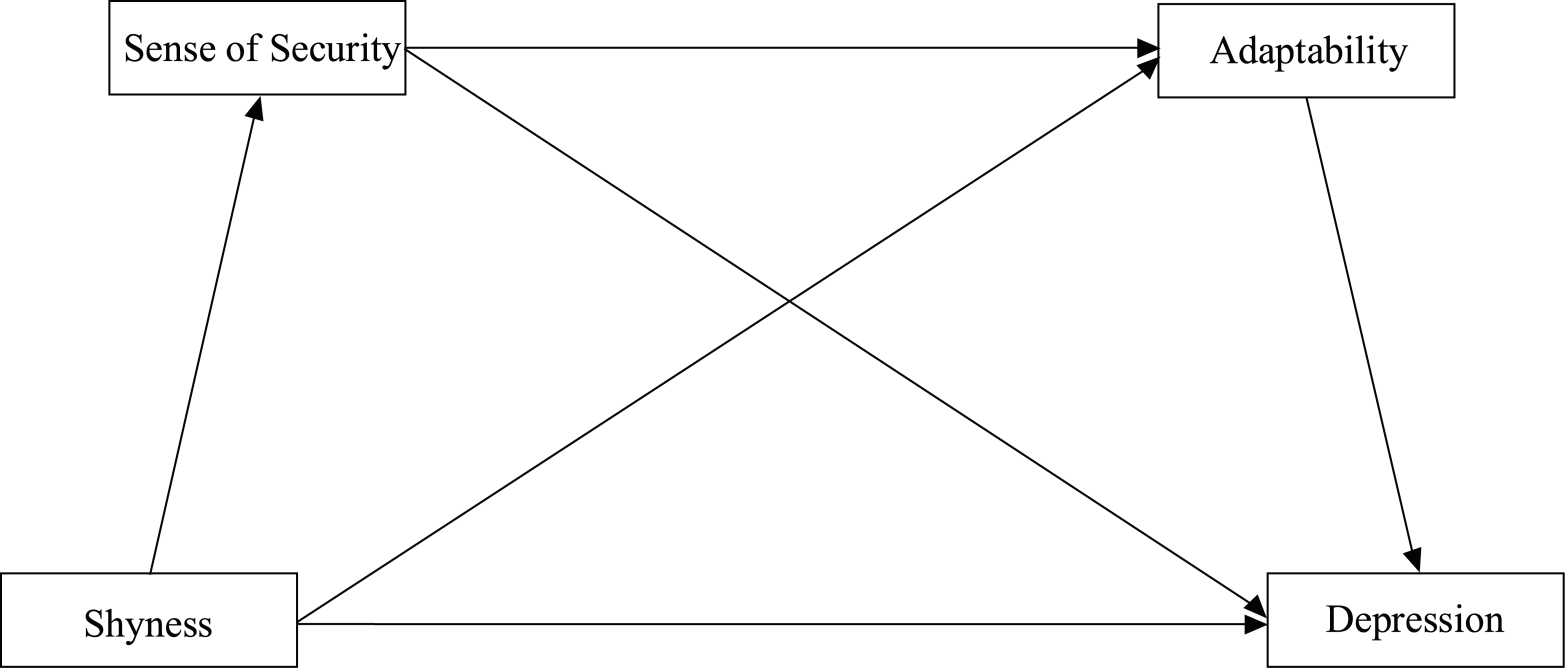
Hypothetical model.

This study, based on the diathesis-stress model, explores the mechanisms linking shyness and depression, focusing on the multiple mediating effects of sense of security and adaptability. The proposed model is shown in Figure 1, with the following hypotheses:

H1: Shyness positively predicts depression.

H2: A sense of security mediates the relationship between shyness and depression.

H3: Adaptation mediates the relationship between shyness and depression.

H4: Both a sense of security and adaptation jointly mediate the relationship between shyness and depression.

## 2 Materials and methods

### 2.1 Participants

This study used a cluster sampling method, recruiting freshmen and sophomores from a university in Shandong Province. A total of 515 questionnaires were distributed. After eliminating invalid questionnaires and those with standardized absolute values greater than |3|, 505 valid data were included in the analysis (M_*age*_ = 19.82, SD = 0.98; Female: 76.83%), resulting in a validity rate of 98.06%. Demographic information for the participants is provided in Table 1. Specifically, (1) in terms of gender, there were 388 females, accounting for 76.8% of the total sample; (2) regarding family location, 179 students lived in urban areas, representing 35.4% of the total, while 99 students lived in town areas, representing 19.6%; (3) in terms of grade, there were 366 sophomores, making up 72.5% of the total sample; (4) regarding only-child status, 302 students came from families with two or more children, comprising 59.8% of the total.

**TABLE 1:** Descriptive statistics of the participants’ basic information (*N* = 505).

Variables	Category	Frequency	Percentage
Gender	Male	117	23.2
	Female	388	76.8
Family location	Urban	179	35.4
	Town	99	19.6
	Rural	227	45.0
Grade	Freshman	139	27.5
	Sophomore	366	72.5
Only child	Yes	203	40.2
	No	302	59.8

### 2.2 Measures

#### 2.2.1 Shyness

The Henderson College Student Shyness Scale, revised by Wang et al. (2009), was employed. This scale includes four dimensions: approval seeking, self-restraint in expression, self-blame, and fear of rejection, with 17 items (e.g., “In social situations, I worry about appearing immature”). The scale uses a 5-point Likert scale, ranging from 1 (completely disagree) to 5 (completely agree). Higher total scores indicate a higher level of shyness. Chen et al. (2018) demonstrated that this scale is applicable to Chinese culture and has strong reliability and validity for assessing shyness among college students.

#### 2.2.2 Sense of security

The Sense of Security Scale, developed by Cong and An (2004), was employed. This scale includes two dimensions: interpersonal security and perceived control. It contains 16 items (e.g., “I never dare to express my opinions proactively”). The scale employs a 5-point Likert scale, ranging from 1 (completely disagree) to 5 (completely agree). Higher total scores indicate a stronger sense of security. Wang et al. (2010) demonstrated that this scale is applicable to Chinese culture and exhibits strong reliability and validity for assessing students’ sense of security.

#### 2.2.3 Adaptability

The College Student Adaptability Scale, developed by Lu (2003), was employed. This scale includes seven dimensions: academic, interpersonal, role, career choice, self-care, overall environmental identification, and physical and mental well-being adaptability. It contains 66 items (e.g., “I feel overwhelmed by my university studies”). The scale employs a 5-point Likert scale, ranging from 1 (completely disagree) to 5 (completely agree). Higher total scores indicate greater adaptability. Zhang (2013) demonstrated that this scale is applicable to Chinese culture and exhibits strong reliability and validity for assessing college student adaptability.

#### 2.2.4 Depression

The Self-Rating Depression Scale, developed by Zung (1965), was employed. It contains 20 items (e.g., “I feel depressed and down”). The scale employs a 4-point scale, ranging from 0 (occasionally) to 3 (continuously). Higher total scores indicate higher levels of depression. Li et al. (2013) found that this scale is applicable to Chinese culture and exhibits strong reliability and validity in their study of suicidal behavior among college students.

### 2.3 Procedure

For data collection, following approval from the administering school, graduate psychology students served as primary examiners and administered the test on a class-by-class basis. Once all participants completed the questionnaire, it was collected immediately, and participants were given a gift. For data analysis, data entry, organization, and preliminary analysis were conducted using SPSS. Structural equation modeling and mediation effect testing were conducted using Mplus.

## 3 Results

### 3.1 Common method bias and reliability testing

Harman’s single-factor test was used to test for common method bias. The results revealed that 31 factors had eigenvalues greater than 1, with the first factor explaining 17.277% of the variance, which is below the critical threshold of 40%, indicating no significant common method bias in this study.

In this study, the Cronbach’s α coefficient and McDonald’s ω coefficient for the shyness scale were 0.868 and 0.870, respectively. the Cronbach’s α coefficients and McDonald’s ω coefficient for the sense of security scale were 0.820 and 0.823, respectively. The Cronbach’s α coefficients and McDonald’s ω coefficient for the adaptability scale were 0.898 and 0.902, respectively. The Cronbach’s α coefficients and McDonald’s ω coefficient for the depression scale were 0.791 and 0.797, respectively. The above results show that the four scales have strong internal consistency.

### 3.2 Preliminary analysis

The correlation coefficients, means, and standard deviations between the variables are shown in Table 2. The results indicate that: (1) shyness is significantly positively correlated with depression, and significantly negatively correlated with both sense of security and adaptation; (2) sense of security is significantly positively correlated with adaptation, and significantly negatively correlated with depression; (3) adaptation is significantly negatively correlated with depression. Furthermore, given the significant correlations between gender, shyness, and sense of security, as well as between grade, shyness, and depression, gender and grade will be included as control variables in subsequent analyses to control for their potential confounding effects.

**TABLE 2:** Descriptive statistics and correlation analysis of research variables.

Variables	*M* ± SD	1	2	3	4	5	6	7	8
1. Gender	–	–	–	–	–	–	–	–	–
2. Age	19.82 ± 0.98	−0.112^*^							
3. FL	–	−0.073	0.275^***^						
4. Grade	–	−0.044	0.584^***^	0.086					
5. Only child	–	0.153^***^	0.142^**^	0.518^***^	0.101^*^				
6. Shyness	50.12 ± 10.16	0.101^*^	−0.079	0.034	−0.196^***^	0.022			
7. SS	53.43 ± 10.22	−0.100^*^	0.060	−0.072	0.019	−0.087	−0.643^***^		
8. Adaptability	232.52 ± 24.67	−0.083	0.055	−0.076	0.079	−0.069	−0.575^***^	−0.588^***^	
9. Depression	36.66 ± 6.76	0.013	0.079	0.066	0.095^*^	0.073	0.476^***^	0.609^***^	−0.601^***^

*p < 0.05,

**p < 0.01,

***p < 0.001. FL, family location; SS, sense of security.

### 3.3 Relationships among variables: a multiple mediation model

After controlling for gender and grade, a structural equation model was developed with shyness as the independent variable, sense of security and adaptability as mediators, and depression as the dependent variable. The model exhibited favorable fit indices (χ^2^/df = 2.942, RMSEA = 0.062, SRMR = 0.032, CFI = 0.991, and TLI = 0.973).

**FIGURE 2:**
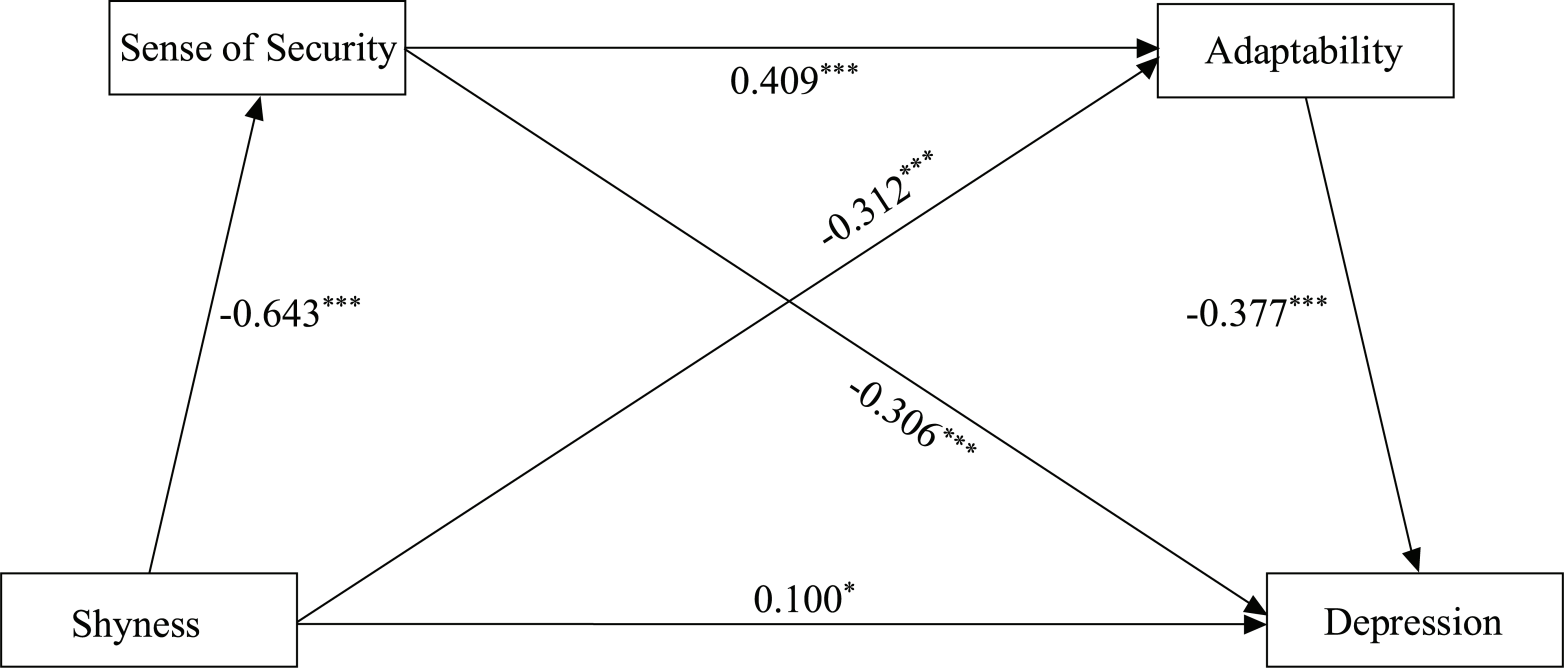
Model results (standardized). *p < 0.05, ***p < 0.001.

Regarding the direct effect, the results (Figure 2) showed that: (1) shyness significantly negatively both predicted sense of security and adaptability (β = −0.643, *P* < 0.001; β = −0.312, *P* < 0.001), and significantly positively predicted depression (β = 0.100, *P* < 0.05); (2) sense of security significantly positively predicted adaptability (β = 0.409, *P* < 0.001), and significantly negatively predicted depression (β = −0.306, *P* < 0.001); (3) adaptability significantly negatively predicted depression (β = −0.377, *P* < 0.001). Hypothesis 1 was supported.

Regarding the indirect effect, the results (Table 3) indicate that: (1) sense of security significantly mediates the relationship between shyness and depression (β = 0.197, 95% CI: [0.126, 0.268]); (2) adaptability significantly mediates the relationship between shyness and depression (β = 0.118, 95% CI: [0.060, 0.176]); (3) sense of security and adaptability significantly jointly mediate the relationship between shyness and depression (β = 0.099, 95% CI: [0.054, 0.144]). Hypotheses 2, 3, and 4 were supported.

**TABLE 3:** Mediation effect test.

		95% CI	
Path	Estimate	Low	Up
Shyness → SS → depression	0.197	0.126	0.268
Shyness → adaptability → depression	0.118	0.060	0.176
Shyness → SS → adaptability → depression	0.099	0.054	0.144

## 4 Discussion

This study examined the underlying mechanisms linking shyness and depression in college students and tested the four proposed hypotheses. The results indicated that: (1) shyness positively predicted depression; (2) sense of security and adaptability not only respectively mediated the relationship between shyness and depression but also played multiple mediating roles in this relationship. This not only revealed the potential relationship between shyness and depression in college students and expanded the diathesis-stress model, but also provided strategies for alleviating depression in a targeted manner. This suggests that college students should offer care and support to each other to enhance the sense of security and reduce the negative environmental perceptions in shy individuals. Additionally, efforts should be made to create a pleasant and relaxed social environment to prevent maladaptation in shy individuals, thereby reducing their internalizing problems such as depression.

### 4.1 Shyness and depression

This study found that shyness in college students positively predicts depression, thereby confirming Hypothesis 1 and supporting previous research (Chen et al., 2018). Shy individuals often have low self-esteem, which leads to the accumulation of negative emotions that can eventually result in depression (Chen et al., 2017; Xu et al., 2024). It is important to note that Chinese culture places high value on interpersonal relationships and frequent social interactions. However, one of the most prominent characteristics of shy individuals is their fear and anxiety in social settings, such as interpersonal interactions (Gao et al., 2016). In contrast to individuals who engage enthusiastically in social interactions, shy individuals often experience greater external pressure. According to the diathesis-stress model, shyness is a vulnerability factor, and individuals who experience significant stress are more prone to depression. Therefore, helping shy college students develop self-awareness and social skills, fostering positive peer interactions (Andrews et al., 2021), and reducing stress can help alleviate depression.

### 4.2 Shyness and depression: sense of security and adaptability as mediators

This study found that sense of security and adaptability mediate the relationship between shyness and depression in college students, supporting and extending previous research (Geng et al., 2021).

First, shyness was found to positively predict depression through a sense of security, which confirms Hypothesis 2. Shy individuals often have fearful expectations about their performance, believing they will perform poorly, which can lower their sense of security (Gao et al., 2016; Han et al., 2016). Previous research has shown that sense of security negatively predicts depression (Fan et al., 2021). According to the diathesis-stress model, shyness, as a susceptibility factor, can lead to increased psychological burden and negative emotions in individuals experiencing chronic sense of insecurity. As negative emotions accumulate, individuals become more likely to develop depression. Therefore, proactive efforts should be made to provide shy individuals with greater sense of security, reduce their stress, and thus alleviate their depression.

Second, shyness was found to positively predict depression through adaptation, which confirms Hypothesis 3. Research has shown that shyness has a negative adaptive value and is a predictor of maladaptation (Poole et al., 2020). For shy college students, adapting to the university’s interpersonal environment is a major source of stress (Gao et al., 2022). Poor interpersonal adaptation can lead to learning and daily life problems, contributing to long-term low mood and, ultimately, depression (Zeng et al., 2024). Therefore, shy individuals should learn more adaptive skills to improve their adaptability, reduce the harm caused by stress, and further reduce negative emotions and depression.

Finally, shyness was found to positively predict depression through both sense of security and adaptation, which confirms Hypothesis 4. Shy individuals experience heightened fear, anxiety, and social evaluation in social situations (Hipson et al., 2019), often resulting in a lower sense of security. Research shows that individuals lacking a sense of security often exhibit poor social adaptability and delayed psychological development (Liu and Liu, 2007). Long-term maladjustment not only severely affects individuals’ physical and mental health (Sun et al., 2023), but also leads to frequent frustration in learning and daily life, further exacerbating stress and anxiety, thus increasing the likelihood of depression.

### 4.3 Limitations and future recommendations

This study has several limitations. First, this study focused on college students, so the results should be generalized to other age groups or working populations with caution. Second, the participants in this study were all from the same university. Future research should expand the sample to include college students from various regions to enhance the generalizability of the results. Third, the self-reported data collection method used in this study may have introduced social desirability bias. Future research could integrate multiple methods, such as experimental approaches, physiological indicators, and longitudinal design, to enable more objective investigations. Finally, this study found that sense of security and adaptability mediate the relationship between shyness and depression. However, this does not rule out the possibility of other mediating or moderating variables between shyness and depression. Future research could further explore the relationships between shyness and depression, including factors such as loneliness and emotion regulation (e.g., cognitive reappraisal and expressive suppression).

Moreover, our study is set within a Chinese cultural context, where shyness is commonly perceived as a negative personality trait, such as awkwardness in public, difficulty expressing oneself, and retreating from significant challenges. However, recent research has demonstrated that shyness is not always a negative trait. For example, children with positive shyness may display emotional regulation processes at the neural level (Poole and Schmidt, 2020), as well as stronger self-awareness, intrinsic motivation, and observational skills (Lawson et al., 2023; Jung et al., 2025). Additionally, cultural perceptions of shyness vary across societies. In Sweden, for example, shyness is considered a desirable trait. This societal consensus can significantly influence the adaptive development of shy individuals. Therefore, shyness should not be viewed solely through a risk-based framework or confined to a single cultural context. Future research should examine both the positive and negative effects of shyness in a multicultural context to better understand its relationship with depression, offering more comprehensive theoretical and practical guidance for the adaptive development of shy individuals.

## 5 Conclusion

This study identifies the underlying mechanisms linking shyness and depression in college students, focusing specifically on the mediating roles of sense of security and adaptation. These findings confirm our proposed hypotheses and extend the diathesis-stress model. The findings are as follows: first, sense of security and adaptability each mediate the relationship between shyness and depression. Second, sense of security and adaptability play multiple mediating roles in the relationship between shyness and depression.

## Data Availability

The data that support the findings of this study are openly available in OSF at https://osf.io/mgz4e/?view_only=b1e37e9138eb419abb8aab8670ecf381. Further inquiries can be directed to the corresponding author.
